# Detailed analysis of learning phases and outcomes in robotic and endoscopic thyroidectomy

**DOI:** 10.1007/s00464-024-11247-2

**Published:** 2024-09-16

**Authors:** Jia-Fan Yu, Wen-Yu Huang, Jun Wang, Wei Ao, Si-Si Wang, Shao-Jun Cai, Si-Ying Lin, Chi-Peng Zhou, Meng-Yao Li, Xiao-Shan Cao, Xiang-Mao Cao, Zi-Han Tang, Zhi-hong Wang, Surong Hua, Wen-Xin Zhao, Bo Wang

**Affiliations:** 1 Department of Thyroid Surgery, Fujian Medical University Union Hospital, Fuzhou, FJ China; 2 Clinical Research Center for Precision Management of Thyroid Cancer of Fujian Province, Fuzhou, FJ China; 3 Department of ENT, Shaxian General Hospital, Sanming, FJ China; 4 Department of General Surgery, Ninghua General Hospital, Sanming, FJ China; 5 Department of Thyroid Surgery, the First Affiliated Hospital of China Medical University, Shenyang, LN China; 6 Department of General Surgery, Peking Union Medical College, Peking, China; 7 Department of Surgery, Massachusetts General Hospital, Harvard Medical School, Boston, MA USA

**Keywords:** daVinci robotic thyroidectomy, Endoscopic thyroidectomy, Learning curves, Bilateral axillo-breast approach (BABA), Minimally invasive surgery

## Abstract

**Background:**

Thyroid surgery has undergone significant transformation with the introduction of minimally invasive techniques, particularly robotic and endoscopic thyroidectomy. These advancements offer improved precision and faster recovery but also present unique challenges. This study aims to compare the learning curves, operational efficiencies, and patient outcomes of robotic versus endoscopic thyroidectomy.

**Methods:**

A retrospective cohort study was conducted, analyzing 258 robotic (da Vinci) and 214 endoscopic thyroidectomy cases. Key metrics such as operation duration, drainage volume, lymph node dissection outcomes, and hypoparathyroidism incidence were assessed to understand surgical learning curves and efficiency.

**Results:**

Robotic thyroidectomy showed a longer learning curve with initially longer operation times and higher drainage volumes but superior lymph node dissection outcomes. Both techniques were safe, with no permanent hypoparathyroidism or recurrent laryngeal nerve damage reported. The study delineated four distinct stages in the robotic and endoscopic surgery learning curve, each marked by specific improvements in proficiency. Endoscopic thyroidectomy displayed a shorter learning curve, leading to quicker operational efficiency gains.

**Conclusion:**

Robotic and endoscopic thyroidectomies are viable minimally invasive approaches, each with its learning curves and efficiency metrics. Despite initial challenges and a longer learning period for robotic surgery, its benefits in complex dissections may justify specialized training. Structured training programs tailored to each technique are crucial for improving outcomes and efficiency. Future research should focus on optimizing training protocols and increasing accessibility to these technologies, enhancing patient care in thyroid surgery.

## Introduction

The integration of advanced surgical technologies has revolutionized the field of thyroid surgery, offering new avenues through robotic and endoscopic techniques. These technologies have not only enhanced surgical precision but have also improved esthetic outcomes and reduced recovery times, making them increasingly popular worldwide. Despite their rapid adoption, a comprehensive understanding of their learning curves and operational efficiencies remains critical.

Robotic thyroidectomy, facilitated by advanced robotic systems, offers unmatched dexterity and control, allowing for precise dissections with minimal damage to surrounding tissues and potentially better patient outcomes [1, 2]. On the other hand, endoscopic thyroidectomy employs minimal access techniques to achieve smaller incisions, reducing postoperative discomfort and speeding up patient recovery [3]. Both methodologies aim to mitigate the physical impact of surgery, aspiring to meet or exceed the results of conventional thyroidectomy.

However, the adoption of these innovative techniques is not without its challenges. The learning curve for mastering these technologies significantly influences operation duration and the overall success of the procedures [4]. Factors such as operation duration, drainage volume, and postoperative hypoparathyroidism rates are critical for evaluating the effectiveness and safety of robotic and endoscopic thyroidectomies [5].

Previous studies have often segmented the learning curve based solely on operation time, without highlighting the phased, multi-stage nature of mastering these techniques [6, 7]. This study delves into the distinctions between robotic and endoscopic thyroidectomy techniques, with a particular focus on their learning curves, operational efficiency, and patient outcomes. By comparing operation duration, drainage volume, lymph node dissection outcomes, and hypoparathyroidism incidence, we aim to enrich the current discourse on these minimally invasive surgical options. Our goal is to enrich the dialogue surrounding these minimally invasive techniques and provide a detailed analysis of their impacts on current surgical practices. Through this analysis, we aim to highlight the inherent advantages and necessary considerations for the adoption of these cutting-edge surgical technologies, thus supporting their integration into mainstream surgical protocols.

## Methods

### Study design and participant criteria

This retrospective cohort study analyzed 472 thyroidectomy procedures, marking the initial adoption phase of innovative surgical techniques at our institution. The procedures comprised 258 robotic thyroidectomies, performed using the da Vinci system between March 2018 and July 2023, and 214 endoscopic thyroid surgeries, conducted from October 2022 to July 2023. Notably, both surgical categories were spearheaded by surgeons at the outset of mastering their respective techniques—Dr. Zhao embarked on learning robotic surgery, while Dr. Wang initiated his journey with endoscopic surgery around the same periods, without any crossover between the two techniques. Eligible participants were aged 13–70, presenting with benign or malignant thyroid nodules not exceeding 5 cm in diameter. Patients were included regardless of central compartment lymph node involvement or distant metastasis presence. Exclusions were made for cases with tumor invasion into surrounding structures, lateral lymph node metastasis, previous neck surgeries, or contraindications to general anesthesia. This retrospective cohort study was conducted in accordance with the ethical standards of the institutional and/or national research committee and with the 1964 Helsinki declaration and its later amendments or comparable ethical standards. Given the retrospective analysis of de-identified data, IRB approval and written informed consent from participants were not required for this study.

### Preoperative assessment

Patients underwent comprehensive preoperative evaluations, including neck and chest CT [8], high-resolution ultrasonography, and fine-needle aspiration biopsies, with optional testing for BRAF V600E mutations and RET gene rearrangements. Preoperative laryngoscopy was performed to assess vocal cord mobility and tailor surgical approaches.

### Surgical techniques

#### Robotic surgery technique

The robotic thyroidectomy was performed using the da Vinci Surgical System, with surgeons alternating between the Si and Xi models depending on availability and specific case requirements. The Bilateral Axillo-Breast Approach (BABA) was the chosen surgical pathway, as detailed in our prior publications [9, 10]. This approach was complemented by intraoperative nerve monitoring [11] and the innovative use of preoperative carbon nanoparticle injections, aimed at enhancing the visualization of lymph nodes and parathyroid glands [12]. A dedicated surgical technician provided crucial support with instrument exchanges, while neck needle retractors were employed to facilitate surgical access and maneuverability as necessary. Dr. Zhao, having completed over 2000 endoscopic thyroid surgeries, underwent training to lead surgeries using the da Vinci robot.

#### Endoscopic surgery technique

Endoscopic thyroidectomy adopted a trans-areolar approach, involving strategic incisions at the bilateral areolas and the anterior chest wall. This technique necessitated a collaborative effort, featuring a lead surgeon and assistants responsible for managing the scope and surgical retractor. For surgeries involving the right thyroid lobe, MiniLap was incorporated to enhance surgical precision and control [13, 14]. Apart from the establishment of the surgical working space, the endoscopic method mirrored the robotic technique in terms of procedural steps and objectives. However, due to constraints associated with Diagnostic Related Group (DRG) policies, the majority of patients did not receive carbon nanoparticle injections or benefit from intraoperative nerve monitoring technology. Dr. Wang is a high-volume thyroid surgeon with over 10 years of experience.

#### Management of parathyroid glands

Routine exploration of the parathyroid glands is part of our standard procedure to ensure their preservation and function. During surgery, surgeons assess the parathyroid glands to decide whether to preserve them in situ or perform autotransplantation based on their viability. Non-viable parathyroid glands identified in situ or ex vivo via near-infrared imaging were autotransplanted [15]. Viability assessments and postoperative care included intravenous and oral calcium supplementation until serum parathyroid hormone levels normalized.

### Statistical analysis

Data were analyzed using SPSS version 29.0, focusing on operative times, complication rates, lymph node dissections, and key biochemical markers. Comparative analyses employed ANOVA and Chi-square tests for continuous and categorical variables, respectively, with significance established at *p* < 0.05. Additionally, the Cumulative Sum (CUSUM) analysis was employed to identify specific turning points or shifts in performance metrics for each indicator. This method is particularly useful for detecting improvements in surgical proficiency over time, marking where surgeons transition from the learning phase to a phase characterized by increased efficiency and stability in outcomes.

## Results

### Demographic and pathological profiles in thyroidectomy

Our study included 472 patients undergoing thyroidectomy, comprising 258 cases in the da Vinci robotic group, which utilized both Si and Xi robot models (123 Si vs. 135 Xi), and 214 cases in the endoscopic group. The da Vinci cohort exhibited a younger mean age of 36.6 ± 8.1 years compared to the endoscopic group's 41.3 ± 11.2 years, marking a statistically significant age difference (*p* < 0.001). Pathologically, the da Vinci group had a smaller mean tumor diameter (0.7 ± 0.4 cm) compared to the endoscopic group (0.9 ± 0.8 cm), with this difference being statistically significant (*p* < 0.001). There were no significant differences observed in the incidence of Hashimoto's thyroiditis, multifocality, intraductal dissemination, and extrathyroidal extension, indicating similar pathological profiles across the cohorts.

### Surgical outcomes

Our study highlighted distinct postoperative biochemical control and surgical efficiency between the robotic and endoscopic thyroidectomy groups. On the first postoperative day, the robotic (da Vinci) group experienced a higher incidence of transient hypoparathyroidism (44.6%, 115 cases) and notable differences in parathyroid hormone (PTH) and calcium levels compared to the endoscopic group (24.8%, 53 cases), with significant disparities in PTH (*p* < 0.001) and calcium levels (*p* < 0.001). This trend of biochemical differences extended into the first week post-operation, with significant differences in PTH (*p* = 0.007) and calcium levels (*p* < 0.001). However, by the first month, while calcium levels continued to significantly differ (*p* = 0.001), PTH levels aligned between the groups (*p* = 0.729), suggesting an eventual stabilization of parathyroid function management across both surgical techniques (Tables 1 and 2).

**Table 1:** Demographics and surgical outcomes of daVinci and endoscopic patients

Parameter / Group	daVinci	Endoscopic	*p*-value
Number of cases	258	214	NA
Age (years, Mean ± Std)	36.6 ± 8.1	41.3 ± 11.2	0.000*
Gender (Male/Female)	75/183	39/175	0.008*
Body mass Index (BMI) (Kg/m^2^, Mean ± Std)	22.6 ± 3.3	23.1 ± 3.7	0.090
da Vinci robot Model (Si/Xi)	123/135	NA	0.000*
Surgical approach (Hemi/Bilateral)	226/32	165/49	0.004*
Pathological diagnosis	Benign: 1, PTC: 257 FTC 1	Benign: 14 NIFTP: 3 PTC 197	0.001*
PTC variation	Classic 234 Follicular 36	Classic 154 Follicular 63 Tall Cell 1	0.001*
Hashimoto's thyroiditis (Y, %)	69(26.7%)	53(24.7%)	0.832
Multifocality (Y, %)	93(36.0%)	70(32.7%)	0.508
Intraductal Dissemination (N)	7	1	0.128
Extrathyroidal extension (N, %)	70(27.7%)	42(21.1%)	0.135
Tumor diameter (cm, Mean ± Std)	0.7 ± 0.4	0.9 ± 0.8	0.001*
Case of parathyroid transplanted (N, %)	209(81.0%)	175(81.8%)	0.925
Parathyroid transplant number (Mean ± Std)	1.1 ± 0.8	1.0 ± 0.7	0.123
PTH Pre OP (pmol/L, Mean ± Std)	4.3 ± 1.9	4.5 ± 1.9	0.210
CA Pre OP (mmol/L, Mean ± Std)	2.3 ± 0.1	2.3 ± 0.1	0.275
PTH 1 Day Post OP (pmol/mL, Mean ± Std)	1.9 ± 1.3	2.5 ± 1.5	0.000*
Calcium 1 Day Post OP (mmol/L, Mean ± Std)	2.3 ± 0.1	2.3 ± 0.1	0.184
PTH decrease day 1 (pmol/mL, Mean ± Std)	2.3 ± 1.8	2.0 ± 1.6	0.060
Calcium decrease day 1 (mmol/L, Mean ± Std)	0.0 ± 0.1	0.0 ± 0.1	0.701
PTH 1 week post OP (pmol/mL, Mean ± Std)	3.1 ± 2.2	2.5 ± 1.9	0.007*
Calcium 1 week post OP (mmol/L, Mean ± Std)	2.3 ± 0.2	2.4 ± 0.1	0.001*
PTH 1 month post OP (pmol/mL, Mean ± Std)	4.2 ± 1.9	4.3 ± 2.4	0.729
Calcium 1 Month Post OP (mmol/L, Mean ± Std)	2.3 ± 0.1	2.4 ± 0.1	0.001*
Hypoparathyroidism on day 1 post OP (N, %)	115(44.6%)	53(24.8%)	0.001*
Hypoparathyroidism on 1 month post OP (N, %)	29(11.2%)	24(11.2%)	1.000
N Stage (N1, %)	148(57.6%)	103(52.3%)	0.140
Number of lymph nodes with metastasis (Mean ± Std)	2.0 ± 3.0	1.5 ± 2.3	0.037*
Number of lymph nodes harvested (Mean ± Std)	12.2 ± 6.3	10.1 ± 5.7	0.000*
Operation duration (min, Mean ± Std)	151.6 ± 34.2	96.7 ± 25.9	0.000*
Drainage day 1 (ml, Mean ± Std)	84.8 ± 34.7	56.4 ± 24.2	0.000*
Drainage volume (ml, Mean ± Std)	161.6 ± 64.3	111.4 ± 44.5	0.000*
Postoperative hospitalization days (Mean ± Std)	2.7 ± 0.8	2.6 ± 0.7	0.530

In our cohort, the reference ranges are PTH: 1.3 to 9.3 pmol/L and calcium: 2.1 to 2.7 mmol/L

**Table 2:** Endoscopic and robotic groups CUSUM analysis of different indicators

Indicators	daVinci group		Endoscopic group	
	Max deviation	Change point	Max deviation	Change point
Operation duration (min)	1625.12	40	307.27	30
Drainage volume day 1 (ml)	1043.78	157	491.60	48
Total drainage volume (ml)	2019.57	157	381.93	115
Parathyroid hormone (PTH) level 1 day post-Op (pmol/mL)	39.33	164	27.35	147
Calcium level 1 Day Post-Op (mmol/L)	− 3.85	116	− 1.44	52
PTH level 1 week Post-Op (pmol/mL)	14.83	129	− 29.24	130
Calcium LEVEL 1 week Post-Op (mmol/L)	− 2.77	195	3.19	96
PTH level 1 month Post-Op (pmol/mL)	− 22.37	60	20.48	58
Calcium level 1 month Post-Op (mmol/L)	2.55	58	1.70	93
PTH day 1 decrease (pmol/mL)	− 22.40	126	23.01	127
Calcium day 1 decrease (mmol/L)	4.22	113	1.59	119
Hospital stay duration (days)	68.30	88	25.92	79
Postoperative hospital stay duration (days)	32.71	133	− 19.78	57
Number of parathyroid transplants	− 9.88	127	12.47	81
Number of lymph nodes harvested	− 146.12	84	− 60.18	162

Additionally, the operation duration was markedly longer in the robotic group (mean 151.6 min, SD ± 34.2) compared to the endoscopic group (mean 96.7 min, SD ± 25.9), *p* = 0.001. Postoperative drainage followed a similar pattern, with the robotic group exhibiting significantly higher volumes on the first day (mean 84.8 ml, SD ± 34.7) and overall (mean 161.6 ml, SD ± 64.3) than the endoscopic group (first-day mean 56.4 ml, SD ± 24.2; total mean 111.4 ml, SD ± 44.5), *p* = 0.001. Despite these differences, the length of postoperative hospital stays was comparable between the groups, underscoring the efficiency of postoperative care protocols. The robotic group also demonstrated a superior capability in lymph node dissection, with a significantly higher number of lymph nodes harvested (mean 12.2, SD ± 6.3) than in the endoscopic group (mean 10.1, SD ± 5.7), *p* = 0.001, indicating a nuanced advantage in surgical precision for complex thyroid cases.

### Mastering thyroid surgery through CUSUM milestones: a structured approach

**Fig. 1:**
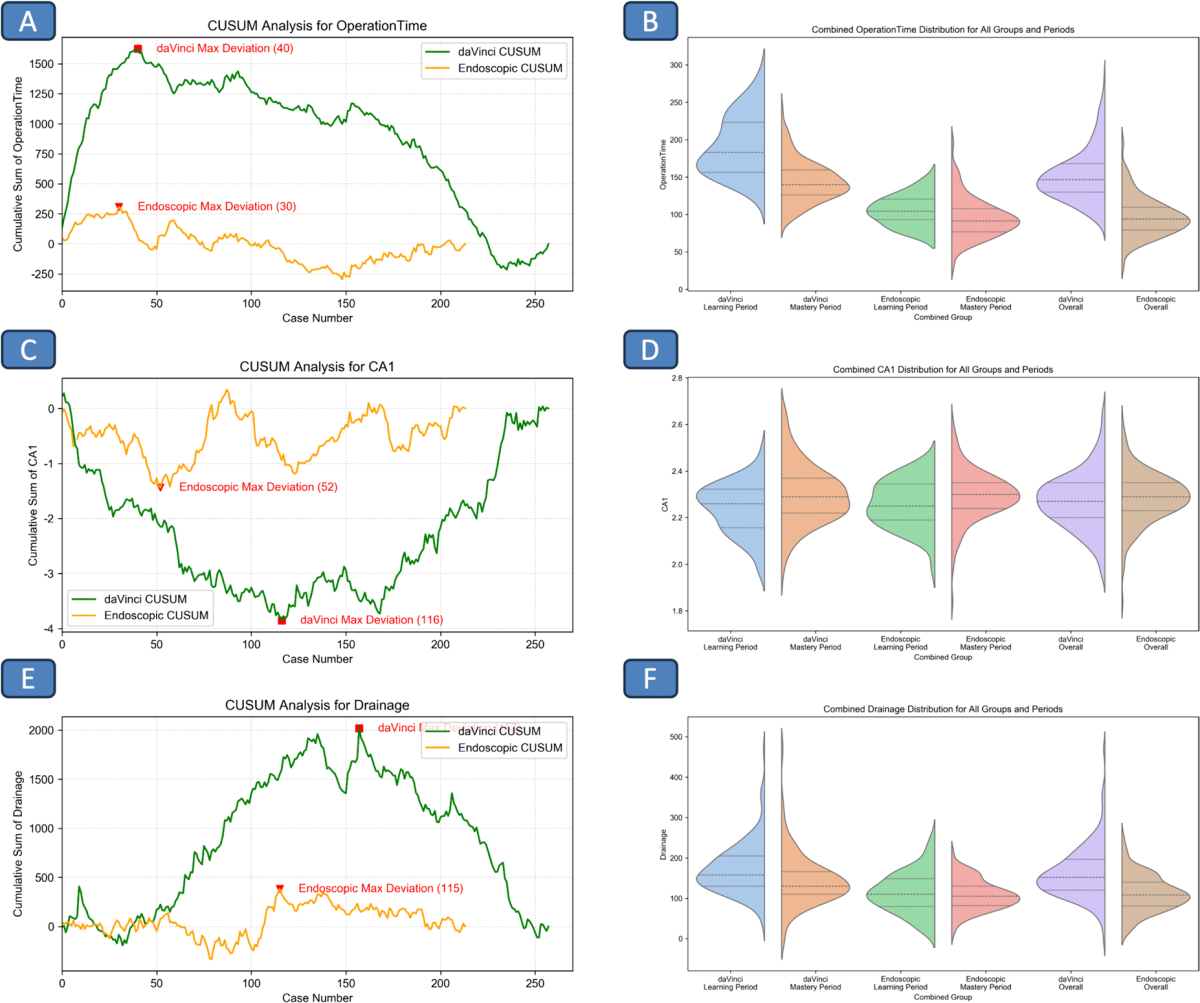
Comparative CUSUM analysis across key metrics for daVinci and endoscopic surgical cohorts, featuring violin plots to highlight data distributions before and after change points. Figure 1**A** & **B**: In-depth CUSUM analysis of operation durations for daVinci versus endoscopic groups, presented with Pre- and Post-Change point violin plots for a deeper visual insight. This comparison aims to elucidate the nuances in surgery times between the two techniques. Figure 1**C** & **D**: CUSUM analysis of day 1 post-operative blood calcium levels for both daVinci and endoscopic surgical groups, augmented with violin plots that reveal variability and change points. This analysis offers a detailed look into the immediate post-operative outcomes and their fluctuations. Figure 1**E** & **F**: Targeted CUSUM analysis on total drainage volumes across daVinci and endoscopic patient sets, with violin plots that illustrate before and after change point distributions. This segment focuses on critical early post-operative biochemical markers, highlighting differences and trends between the surgical approaches

The progression of surgeons mastering thyroid surgery is elucidated through the Cumulative Sum (CUSUM) analysis, applied to both endoscopic and robotic (da Vinci) techniques. Drawing from extensive data in Table 3 and visualizations in Fig. 1, this analysis divides the learning curve into four critical phases: Foundation and Adaptation, Innovation and Enhancement, Functional Focus, and Anatomical Precision.

**Table 3:** Comparative analysis of surgical proficiency

Parameter / Group	daVinci group					Endoscopic group				
	Foundation and adaptation phase	Innovation and enhancement phase	Functional focus phase	Anatomical precision phase	*p*-value	Foundation and adaptation phase	Innovation and enhancement phase	Functional focus phase	Anatomical precision phase	*p*-value
Case number (N)	40	76	41	101	NA	30	22	63	99	NA
Gender (Male/Female)	10/30	29/47	9/32	27/74	0.200	4/26	3/19	15/48	17/82	0.535
DaVinci robot model (Si/Xi)	28/12	28/48	17/24	50/51	0.006*	NA	NA	NA	NA	NA
Age (Years, Mean ± Std)	35.30 ± 8.71	36.66 ± 8.00	35.68 ± 7.44	37.43 ± 8.21	0.459	37.80 ± 6.24	41.55 ± 14.36	44.05 ± 11.33	40.56 ± 11.31	0.067
BMI (Mean ± Std)	22.52 ± 3.70	22.93 ± 3.29	22.62 ± 3.11	22.36 ± 3.16	0.719	22.34 ± 2.14	23.94 ± 7.48	23.73 ± 3.35	22.83 ± 3.03	0.199
Operation duration (min, Mean ± Std)	191.90 ± 39.19	145.80 ± 26.88	150.76 ± 28.32	140.24 ± 27.20	0.000*	105.27 ± 19.77	87.68 ± 34.27	95.22 ± 24.75	97.05 ± 25.69	0.103
HospitalStay Hospitalization Days (Mean ± Std)	8.10 ± 3.13	7.74 ± 2.17	6.41 ± 2.27	7.13 ± 3.00	0.018*	6.93 ± 1.80	8.50 ± 5.70	7.86 ± 2.85	7.58 ± 3.08	0.353
Postoperative Hospitalization Days (Mean ± Std)	3.05 ± 0.93	2.82 ± 0.80	2.49 ± 0.71	2.46 ± 0.56	0.000*	2.23 ± 0.50	2.32 ± 0.48	2.86 ± 0.64	2.65 ± 0.73	0.000*
Drainage Volume (ml, Mean ± Std)	163.05 ± 68.10	181.28 ± 58.15	165.59 ± 62.79	144.69 ± 64.01	0.002*	113.50 ± 36.29	109.27 ± 48.19	116.49 ± 56.84	107.99 ± 36.54	0.679
Postoperative day 1 Drainage Volume (ml, Mean ± Std)	83.97 ± 45.15	93.39 ± 29.10	93.24 ± 39.31	75.28 ± 29.39	0.002*	66.77 ± 28.51	62.05 ± 30.51	53.29 ± 25.54	53.89 ± 19.06	0.030*
Parathyroid Transplant Number (Mean ± Std)	1.10 ± 0.81	1.04 ± 0.70	1.24 ± 0.86	1.15 ± 0.79	0.574	1.13 ± 0.51	1.18 ± 0.50	0.97 ± 0.69	0.98 ± 0.76	0.434
Total Number of Lymph Nodes Harvested (Mean ± Std)	10.03 ± 5.94	11.87 ± 6.35	13.61 ± 4.78	12.61 ± 6.84	0.065	10.29 ± 5.14	9.41 ± 5.58	10.37 ± 6.98	10.01 ± 5.16	0.918
Lymph nodes with Metastasis (Mean ± Std)	1.82 ± 2.68	2.33 ± 3.57	2.34 ± 2.57	1.76 ± 2.88	0.542	1.29 ± 1.65	1.27 ± 1.39	1.70 ± 3.23	1.47 ± 2.09	0.841
PTH 1Day Post OP (pmol/L, Mean ± Std)	2.43 ± 1.22	1.97 ± 1.25	2.27 ± 1.70	1.60 ± 1.08	0.002*	2.68 ± 0.97	2.64 ± 0.95	2.59 ± 1.53	2.27 ± 1.71	0.393
PTH Day1 Decreased Post OP (pmol/L, Mean ± Std)	2.45 ± 1.95	2.07 ± 1.49	2.66 ± 1.96	2.37 ± 1.80	0.349	1.86 ± 1.44	1.98 ± 1.61	2.46 ± 2.00	1.83 ± 1.42	0.106
PTH 1Week Post OP (pmol/L, Mean ± Std)	3.21 ± 1.75	2.97 ± 2.00	3.38 ± 1.92	3.00 ± 2.52	0.775	2.55 ± 2.58	2.39 ± 1.41	2.27 ± 1.37	2.66 ± 2.11	0.683
PTH 1Month Post OP (pmol/L, Mean ± Std)	3.57 ± 2.10	4.25 ± 1.76	4.52 ± 1.95	4.04 ± 1.87	0.317	4.51 ± 2.24	4.32 ± 2.25	4.34 ± 2.74	4.03 ± 2.34	0.844
CA 1Day Post OP (pmol/L, Mean ± Std)	2.23 ± 0.11	2.25 ± 0.11	2.28 ± 0.12	2.31 ± 0.13	0.001*	2.27 ± 0.10	2.24 ± 0.10	2.30 ± 0.12	2.29 ± 0.09	0.140
CA Day1 Decreased Post OP (pmol/L, Mean ± Std)	0.08 ± 0.12	0.07 ± 0.11	− 0.01 ± 0.15	0.02 ± 0.12	0.000*	0.02 ± 0.09	0.06 ± 0.12	0.05 ± 0.13	0.02 ± 0.10	0.265
CA 1Week Post OP (pmol/L, Mean ± Std)	2.38 ± 0.10	2.30 ± 0.13	2.25 ± 0.24	2.34 ± 0.14	0.004*	2.37 ± 0.11	2.48 ± 0.09	2.45 ± 0.12	2.37 ± 0.13	0.000*
CA 1Month Post OP (pmol/L, Mean ± Std)	2.40 ± 0.12	2.29 ± 0.11	2.26 ± 0.14	2.31 ± 0.11	0.000*	2.40 ± 0.12	2.35 ± 0.11	2.34 ± 0.14	2.34 ± 0.12	0.260

In our cohort, the reference ranges are PTH: 1.3 to 9.3 pmol/L and calcium: 2.1 to 2.7 mmol/L

**Foundation and adaptation phase:** Surgeons embark on their path to mastery during this phase, with operation times initially decreasing after the 40th case for the da Vinci group and the 30th case for the endoscopic group. At the onset, operation times average at 191.90 ± 39.19 min for the da Vinci group and 105.27 ± 19.77 min for the endoscopic group, setting the stage for skill development. This phase emphasizes the acquisition of foundational knowledge and the ability to adapt to surgical challenges.

**Innovation and enhancement phase:** This phase signifies a shift towards accelerated surgical speed and a focus on preserving function. With growing proficiency, operation times reduce to 140.24 ± 27.20 min for the da Vinci group and 97.05 ± 25.69 min for the endoscopic group. It underscores a surgeon's dedication to technique refinement and patient outcome improvement, marking a period of surgical innovation and enhancement.

**Functional focus phase:** Surgeons reach a milestone in this phase, optimizing post-operative calcium levels to 2.31 ± 0.13 mmol/L for da Vinci and 2.29 ± 0.09 mmol/L for endoscopic procedures by the 116th and 52nd cases, respectively. This focus on gland function post-surgery is paramount for patient recovery, highlighting the necessity of functional preservation. Remarkably, the endoscopic group requires only 44.8% of the cases needed by the robotic group to achieve this optimization.

**Anatomical precision phase:** The journey culminates with a notable decrease in total drainage volume, demonstrating surgical precision. The endoscopic group reaches this milestone by the 115th case, while the robotic group follows at the 157th case, with volumes decreasing to 144.69 ± 64.01 ml for da Vinci and 107.99 ± 36.54 ml for endoscopic procedures. This phase illustrates the surgeons' ability to minimize trauma and optimize patient care, achieving an elevated level of surgical precision and efficiency.

## Discussion

Recent explorations into the learning curves of robotic and endoscopic thyroidectomy have intensified, with studies increasingly leveraging quantifiable metrics to assess both proficiency and safety. Zhang DQ et al. [16] and Liang et al. [17] notably charted the robotic thyroidectomy learning curve through distinct phases that mirror our observations, initially characterized by longer operation times which gradually taper off as surgeons accumulate hands-on experience. This phased approach to dissecting the learning curve resonates with our findings, reinforcing the critical need for focused training during the formative stages of technological adoption. Our analysis further elucidates that while both robotic and endoscopic techniques offer safe and efficacious avenues for minimally invasive thyroid surgery, they each come with their set of challenges and benefits, necessitating a nuanced approach to surgical training and practice.

The extended learning curve seen with robotic thyroidectomy, marked by longer initial operation times and increased postoperative drainage, underscores the complexity and challenges inherent in mastering robotic surgical systems. The addition of an extra operative arm increases the extent of flap dissection, and early stage unfamiliarity with the robotic controls may be the primary reasons for the increased drainage. This observation aligns with the broader literature [18–20], as highlighted by Chen YH et al. [20] and Bae et al. [19], which acknowledges the steep learning curve typically associated with the adoption of robotic systems in surgical practices. Our study advances this discussion by segmenting the learning curve into four detailed stages, providing a granular roadmap to guide surgeons towards achieving proficiency. Such detailed phase analysis offers insights not commonly explored in existing literature, where the learning curve is often considered a singular, undifferentiated trajectory.

In recent years, Sun et al. [18] and You JY et al. [21] have further substantiated the superior lymph node dissection capabilities afforded by robotic thyroidectomy, attributing these outcomes to the enhanced three-dimensional visualization and surgical precision made possible by robotic systems. This finding corroborates our study's results, indicating that continuous advancements in robotic technology are likely to further refine surgical outcomes. The demonstrable benefits in lymph node dissection with robotic surgery underscore the value of investing in specialized training, suggesting that the initial investment in time and learning can culminate in significant improvements in patient care.

Conversely, research by Chong et al. [22] and Zhou D et al. [23] underscores the significant advantages of endoscopic thyroidectomy, such as shorter hospital stays and reduced postoperative discomfort, thereby emphasizing the technique's role in facilitating quicker patient recovery without compromising on safety. This revelation points towards the accessibility of endoscopic techniques for surgeons, potentially requiring a less steep learning curve compared to robotic systems. Nevertheless, the detailed advantage in lymph node dissection achieved through robotic surgery highlights the importance of carefully selecting the surgical approach that best matches the case complexity and aligns with the surgeon's expertise and training.

Our study presents a notable observation regarding the alignment of postoperative parathyroid hormone (PTH) levels between robotic and endoscopic groups one month following surgery. This occurs despite a more frequent occurrence of transient hypoparathyroidism in the robotic group immediately post-procedure. Such findings suggest the long-term preservation of parathyroid function by both robotic and endoscopic methodologies. The early discrepancies might be linked to a higher incidence of parathyroid autotransplantation in the robotic group, where the precision capabilities of the instruments have not been fully utilized during the learning phase. Supporting this, a meta-analysis by de Vries et al. [24] contrasted minimally invasive techniques with traditional thyroidectomy, revealing no significant differences in hypoparathyroidism rates and recurrent laryngeal nerve damage, thus affirming the safety profile identified in our investigation. This comparative analysis underscores that, given adequate training and experience, minimally invasive approaches can yield safety outcomes on par with conventional surgery. The absence of permanent hypoparathyroidism and recurrent laryngeal nerve injuries in both the robotic and endoscopic groups reinforces the safety profile of minimally invasive thyroidectomy techniques. This finding aligns with established benchmarks, highlighting the reliability of these approaches in preserving critical structures and ensuring patient safety during thyroid surgery. [25–27]

The literature's growing acknowledgment of the importance of structured training programs is mirrored in our findings. Echoing Yu Hyeong Won et al. [28], our study advocates for the creation of comprehensive educational frameworks designed to ease the learning curves associated with advanced surgical technologies. Emphasizing technique-specific training, our research aligns with Russell et al. [29] and Qingqing He et.al. [30], calling for educational models tailored to the unique requirements of robotic and endoscopic surgery. Furthermore, Boal et al. [31] and other authors [32, 33] highlight the significance of simulation-based training and virtual reality platforms in diminishing the learning curve associated with robotic thyroidectomy, suggesting such immersive technologies could markedly improve the surgical skill acquisition process. However, it's important to note that while training may expedite mastery over the machinery, it does not necessarily hasten advancement through later stages of surgical proficiency.

This study not only reaffirms findings from previous research but also enhances the dialogue by providing a detailed exploration of the learning curves for robotic and endoscopic thyroidectomy. By juxtaposing these methods and outlining the progression stages for robotic surgery, we offer valuable insights for refining surgical training and initiating new practices.. Moreover, our comprehensive review of surgical outcomes and efficiency metrics contributes to the accumulating evidence favoring the adoption of minimally invasive techniques in thyroid surgery. Our analysis suggests that while both robotic and endoscopic methods offer distinct advantages, the selection between them should be guided by the specific needs of the case, surgeon expertise, and the resources available within the institution [34].

Our study, while providing significant insights into the learning curves and outcomes of minimally invasive thyroidectomy, is not without its limitations. The retrospective design introduces potential selection biases and limits the ability to control for confounding variables, such as the application of nanoparticle injection. However, these factors are controllable and consistent with existing literature [35]. Additionally, the direct comparison of surgical outcomes may be affected by variations in patient demographics, tumor characteristics, and differing levels of surgeon expertise. To build on our work, future research should prioritize prospective studies to enhance the robustness of findings. There is a critical need for longitudinal studies that assess long-term patient satisfaction and the cost-effectiveness of these surgical techniques. Moreover, the development and implementation of innovative training modules, particularly those incorporating simulation-based learning, are essential for reducing the learning curve and advancing proficiency in both robotic and endoscopic thyroidectomy.

## Conclusion

This comparative study of robotic and endoscopic thyroidectomy has systematically examined the differences and similarities in surgical outcomes and operational efficiencies between these two minimally invasive techniques. Our findings reveal that both robotic and endoscopic thyroidectomies are effective in managing thyroid conditions, each offering unique advantages. Robotic surgery is characterized by its precision and enhanced visual capabilities, typically resulting in better lymph node dissection but requiring longer operation times and a steeper learning curve. Conversely, endoscopic surgery is known for its less invasive nature and quicker patient recovery, though it demands high flexibility and presents certain limitations in complex dissections.

Both robotic and endoscopic thyroidectomy techniques possess unique strengths and necessitate specific training programs. The results of this study contribute to optimizing surgical training and improving patient outcomes in thyroid surgery. Ultimately, the choice between robotic and endoscopic thyroidectomy should be guided by the specific clinical scenario, surgeon expertise, and patient preferences. This study underscores the need for tailored surgical approaches to enhance patient outcomes and operational efficiency. Future research should continue to refine these techniques and explore innovative training programs to equip surgeons with the skills needed to effectively utilize both technologies in clinical practice.

## References

[1] Fassari A, Gurrado A, Iossa A, Micalizzi A, Polistena A, Sibio S, Crocetti D, Bononi M, Testini M, Avenia N (2023) Definition of learning curve for thyroidectomy: systematic review on the different approaches. Gland Surgery 12:989 37727342 10.21037/gs-22-730 PMC10506114

[2] Kim K-h, Ji YB, Song CM, Kim E, Kim KN, Tae K (2023) Learning curve of transoral robotic thyroidectomy. Surgical Endoscopy 37:535–543 36002679 10.1007/s00464-022-09549-4

[3] Anuwong A, Ketwong K, Jitpratoom P, Sasanakietkul T, Duh Q-Y (2018) Safety and outcomes of the transoral endoscopic thyroidectomy vestibular approach. JAMA Surg 153:21–27 28877292 10.1001/jamasurg.2017.3366 PMC5833624

[4] Sun H, Zheng H, Wang X, Zeng Q, Wang P, Wang Y (2020) Comparison of transoral endoscopic thyroidectomy vestibular approach, total endoscopic thyroidectomy via areola approach, and conventional open thyroidectomy: a retrospective analysis of safety, trauma, and feasibility of central neck dissection in the treatment of papillary thyroid carcinoma. Surg Endosc 34:268–274 31346748 10.1007/s00464-019-06762-6

[5] Koimtzis G, Stefanopoulos L, Alexandrou V, Tteralli N, Brooker V, Alawad AA, Carrington-Windo E, Karakasis N, Geropoulos G, Papavramidis T (2022) The role of carbon nanoparticles in lymph node dissection and parathyroid gland preservation during surgery for thyroid cancer: a systematic review and meta-analysis. Cancers 14:4016 36011009 10.3390/cancers14164016 PMC9407010

[6] Kim KH, Ji YB, Song CM, Kim E, Kim KN, Tae K (2023) Learning curve of transoral robotic thyroidectomy. Surg Endosc 37:535–543 36002679 10.1007/s00464-022-09549-4

[7] Yu J, Rao S, Lin Z, Pan Z, Zheng X, Wang Z (2019) The learning curve of endoscopic thyroid surgery for papillary thyroid microcarcinoma: CUSUM analysis of a single surgeon’s experience. Surg Endosc 33:1284–1289 30264276 10.1007/s00464-018-6410-y PMC6430754

[8] Zhang D, Fu Y, Zhou L, Wang T, Liang N, Zhong Y, Dionigi G, Kim HY, Sun H (2021) Prevention of non-recurrent laryngeal nerve injury in robotic thyroidectomy: imaging and technique. Surg Endosc 35:4865–4872 33721091 10.1007/s00464-021-08421-1

[9] Wang B, Yu JF, Ao W, Wang J, Guo XY, Li MY, Huang WY, Zhou CP, Yan SY, Zhang LY, Wang SS, Cai SJ, Lin SY, Zhao WX (2024) Optimizing robotic thyroid surgery: lessons learned from an retrospective analysis of 104 cases. Front Endocrinol Lausanne. 15:133 10.3389/fendo.2024.1337322 PMC10867960 38362277

[10] Prete F, Panzera P, De Luca GM, Vittore F, Testini C, Lavermicocca W, Gurrado A and Testini M (2023) Robotic Bilateral Axillo-Breast Approach, In Thyroid Surgery, pp 93–99: Springer International Publishing Cham, New York

[11] Randolph GW, Dralle H, Abdullah H, Barczynski M, Bellantone R, Brauckhoff M, Carnaille B, Cherenko S, Chiang FY, Dionigi G, Finck C, Hartl D, Kamani D, Lorenz K, Miccolli P, Mihai R, Miyauchi A, Orloff L, Perrier N, Poveda MD, Romanchishen A, Serpell J, Sitges-Serra A, Sloan T, Van Slycke S, Snyder S, Takami H, Volpi E, Woodson G (2011) Electrophysiologic recurrent laryngeal nerve monitoring during thyroid and parathyroid surgery: international standards guideline statement. Laryngoscope 121(Suppl 1):S1-16 21181860 10.1002/lary.21119

[12] Zhang D, Fu Y, Dionigi G, Hu Y, Zhang J, Wang T, Xue G, Sun H (2020) A randomized comparison of carbon nanoparticles in endoscopic lymph node dissection via the bilateral areola approach for papillary thyroid cancer. Surg Laparosc Endosc Percutan Tech 30:291–299 32574006 10.1097/SLE.0000000000000793

[13] Zhao W, Wang B, Yan S, Zhang L (2016) Minilaparoscopy-assisted modified neck dissection through bilateral breast approach. VideoEndocrinology 3(2):1–2 10.1089/ve.2015.0038 PMC6961798 32025527

[14] Zhao W, Wang B, Yan S, Zhang L (2015) Minilaparoscopy-assisted hemithyroidectomy and central neck dissection (Level VI) using bilateral breast approach. VideoEndocrinology 2(1):1–2 10.1089/ve.2015.0038 PMC6961798 32025527

[15] Wang B, Weng YJ, Zheng J, Jia-Fan Y, Wang MT, Zhu YF, Wen J, Zhao WX, Wang B (2020) The management of parathyroid in vitro during thyroid surgery. VideoEndocrinology. 7:1–3

[16] Zhang D, Wang C, Sui C, Li K, Yang M, Xue G, Dionigi G, Kim HY, Sun H (2022) Robotic bilateral axillo-breast versus endoscopic bilateral areola thyroidectomy outcomes of 757 patients. Front Endocrinol Lausanne 13:1029845 36743931 10.3389/fendo.2022.1029845 PMC9895782

[17] Liang T-J, Tsai C-Y, Liu S-I, Chen I-S (2021) Multidimensional analyses of the learning curve of endoscopic thyroidectomy. World J Surg 45:1446–1456 33512565 10.1007/s00268-021-05953-4

[18] Sun HX, Gao HJ, Ying XY, Chen X, Li QY, Qiu WH, Yan JQ (2020) Robotic thyroidectomy via bilateral axillo-breast approach: Experience and learning curve through initial 220 cases. Asian J Surg 43:482–487 31402083 10.1016/j.asjsur.2019.07.015

[19] Bae DS, Koo DH (2019) A propensity score-matched comparison study of surgical outcomes in patients with differentiated thyroid cancer after robotic versus open total thyroidectomy. World J Surg 43:540–551 30242457 10.1007/s00268-018-4802-8

[20] Chen YH, Kim HY, Anuwong A, Huang TS, Duh QY (2021) Transoral robotic thyroidectomy versus transoral endoscopic thyroidectomy: a propensity-score-matched analysis of surgical outcomes. Surg Endosc 35:6179–6189 33111192 10.1007/s00464-020-08114-1

[21] You JY, Kim HK, Kim HY, Fu Y, Chai YJ, Dionigi G, Tufano RP (2021) Bilateral axillo-breast approach robotic thyroidectomy: review of a single surgeon’s consecutive 317 cases. Gland Surg 10:1962–1970 34268080 10.21037/gs-21-50 PMC8258871

[22] Chong K-H, Wu M-H, Lai C-W (2020) Comparison of surgical outcome between conventional open thyroidectomy and endoscopic thyroidectomy through axillo-breast approach. Tzu-Chi Med J 32:286 32955515 10.4103/tcmj.tcmj_109_19 PMC7485670

[23] Zhou D, Zhang Z, Dou X, Xia F, Li X (2024) Advances in the assessment of cosmetic outcomes, sensory alteration in surgical areas, and health-related quality of life of endoscopic thyroidectomy. World J Surg Oncol 22:52 38347606 10.1186/s12957-024-03307-7 PMC10863152

[24] de Vries LH, Aykan D, Lodewijk L, Damen JA, BorelRinkes IH, Vriens MR (2021) Outcomes of minimally invasive thyroid surgery–a systematic review and meta-analysis. Front Endocrinol (Lausanne) 12:719397 34456874 10.3389/fendo.2021.719397 PMC8387875

[25] Lorenz K, Raffaeli M, Barczyński M, Lorente-Poch L, Sancho J (2020) Volume, outcomes, and quality standards in thyroid surgery: an evidence-based analysis—European society of endocrine surgeons (ESES) positional statement. Langenbecks Arch Surg 405:401–425 32524467 10.1007/s00423-020-01907-x PMC8275525

[26] Neagoe RM, Cvasciuc IT, Muresan M, Sala DT (2017) Incidental parathyroidectomy during thyroid surgery - risk, prevention and controversies an evidence-based review. Acta Endocrinol (Buchar) 13:467–475 31149218 10.4183/aeb.2017.467 PMC6516554

[27] Choi YS, Shin WY, Yi JW (2021) Single surgeon experience with 500 cases of the robotic bilateral axillary breast approach (BABA) for thyroid surgery using the Da-Vinci Xi system. J Clin Med 10:4048 34575159 10.3390/jcm10184048 PMC8471909

[28] Yu HW, Yi JW, Seong CY, Kim J-k, Bae IE, Kwon H, Chai YJ, Kim S-j, Choi JY, Lee KE (2018) Development of a surgical training model for bilateral axillo-breast approach robotic thyroidectomy. Surg Endosc 32:1360–1367 28842763 10.1007/s00464-017-5816-2

[29] Russell JO, Razavi CR, Garstka ME, Chen LW, Vasiliou E, Kang S-W, Tufano RP, Kandil E (2019) Remote-access thyroidectomy: a multi-institutional North American experience with transaxillary, robotic facelift, and transoral endoscopic vestibular approaches. J Am Coll Surg 228:516–522 30586640 10.1016/j.jamcollsurg.2018.12.005 PMC6424630

[30] Ma Y, Yue T, He Q (2024) Tracheal injury following robotic thyroidectomy: a literature review of epidemiology, etiology, diagnosis, and treatment and 3 case reports. Asian J Surg 47:83–88 37879990 10.1016/j.asjsur.2023.10.039

[31] Boal MW, Anastasiou D, Tesfai F, Ghamrawi W, Mazomenos E, Curtis N, Collins JW, Sridhar A, Kelly J, Stoyanov D (2024) Evaluation of objective tools and artificial intelligence in robotic surgery technical skills assessment: a systematic review. Br J Surg 111:331 10.1093/bjs/znad331 PMC10771126 37951600

[32] Lee CR, Rho SY, Han SH, Moon Y, Hwang SY, Kim YJ, Kang CM (2019) Comparison of training efficacy between custom-made skills simulator (CMSS) and da Vinci skills simulators: a randomized control study. World J Surg 43:2699–2709 31399794 10.1007/s00268-019-05108-6

[33] Razavi CR, Tanavde V, Shaear M, Richmon JD, Russell JO (2020) Simulations and simulators in head and neck endocrine surgery. Ann Thyroid 5(3):1–6 10.21037/aot.2020.03.03 PMC7213387 32395699

[34] Park YM, Kim DH, Moon YM, Lim JY, Choi EC, Kim SH, Holsinger FC, Koh YW (2019) Gasless transoral robotic thyroidectomy using the DaVinci SP system: feasibility, safety, and operative technique. Oral Oncol 95:136–142 31345381 10.1016/j.oraloncology.2019.06.003

[35] Chen Y, Zhang S, Miao K, Li J (2024) Evaluating the effectiveness of dual dye combination of indocyanine green and carbon nanoparticles with parathyroid hormone test in preserving parathyroid gland during papillary thyroid cancer surgery: a single-center retrospective cohort study. Updates Surg 76:1063–1071 38507176 10.1007/s13304-024-01804-8 PMC11130042

